# Supplementary material for: Identification and Functional Characterization of Novel MYC-Regulated Long Noncoding RNAs in Group 3 Medulloblastoma
Source: Cancers (Basel). 2021 Jul 30;13(15):3853. doi: 10.3390/cancers13153853 (PMC8345409; doi:10.3390/cancers13153853)
Supplement: Supplementary file 1 [file cancers-13-03853-s001.zip › Supplementary Materials/Original Images for Blots.pdf]

**Fig. 1**

**A**

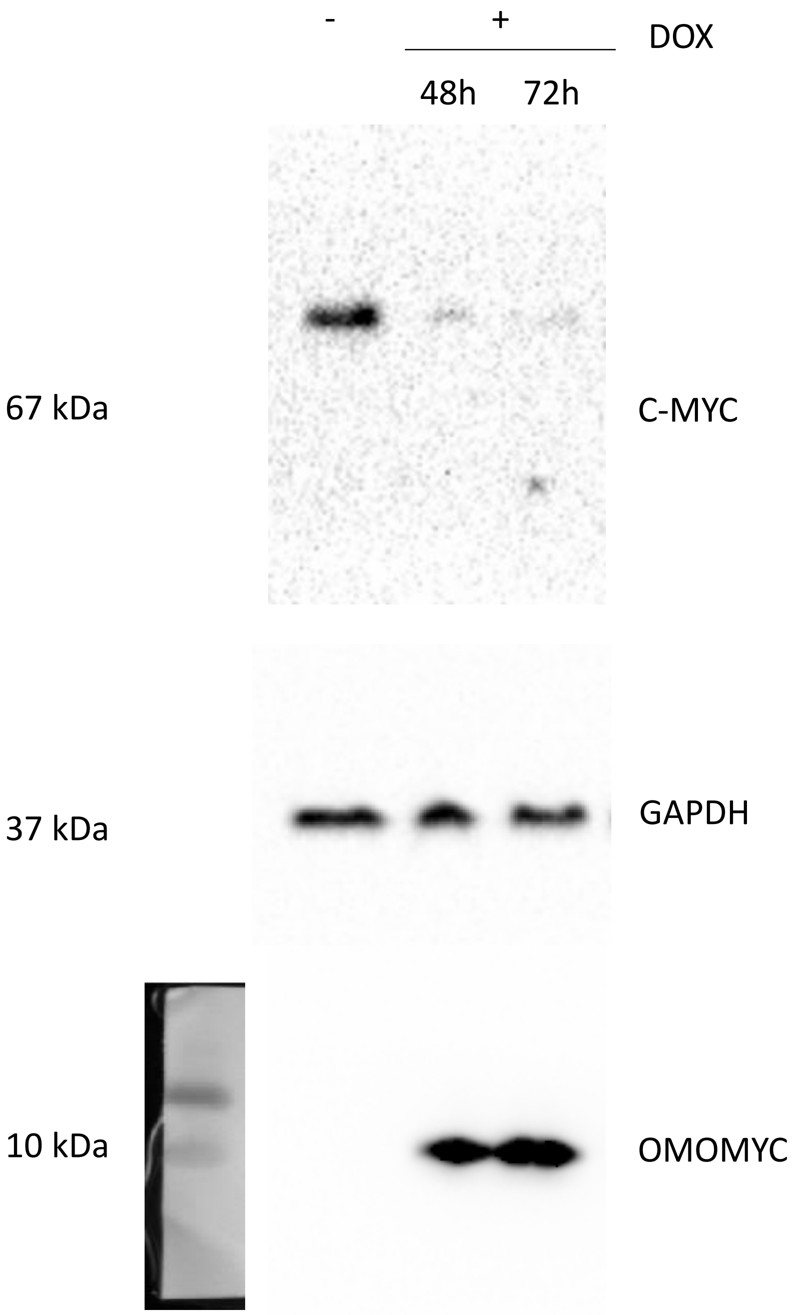


| | GAPDH | OMOMYC | C-MYC | OMOMYC/GAPDH | C-MYC/GAPDH | FC OMOMYC | FC C-MYC |
|---|---|---|---|---|---|---|---|
| DOX - | 875457 | 232933 | 1545355 | 0.266070178 | 1.765198062 | 0.01302679 | 1 |
| DOX + 48h | 644303 | 11239345 | 215440 | 17.44419163 | 0.334376838 | 0.854067251 | 0.189427 |
| DOX + 72h | 706857 | 14437445 | 200509 | 20.42484548 | 0.283662749 | 1 | 0.160697 |

# Fig. 10

## B

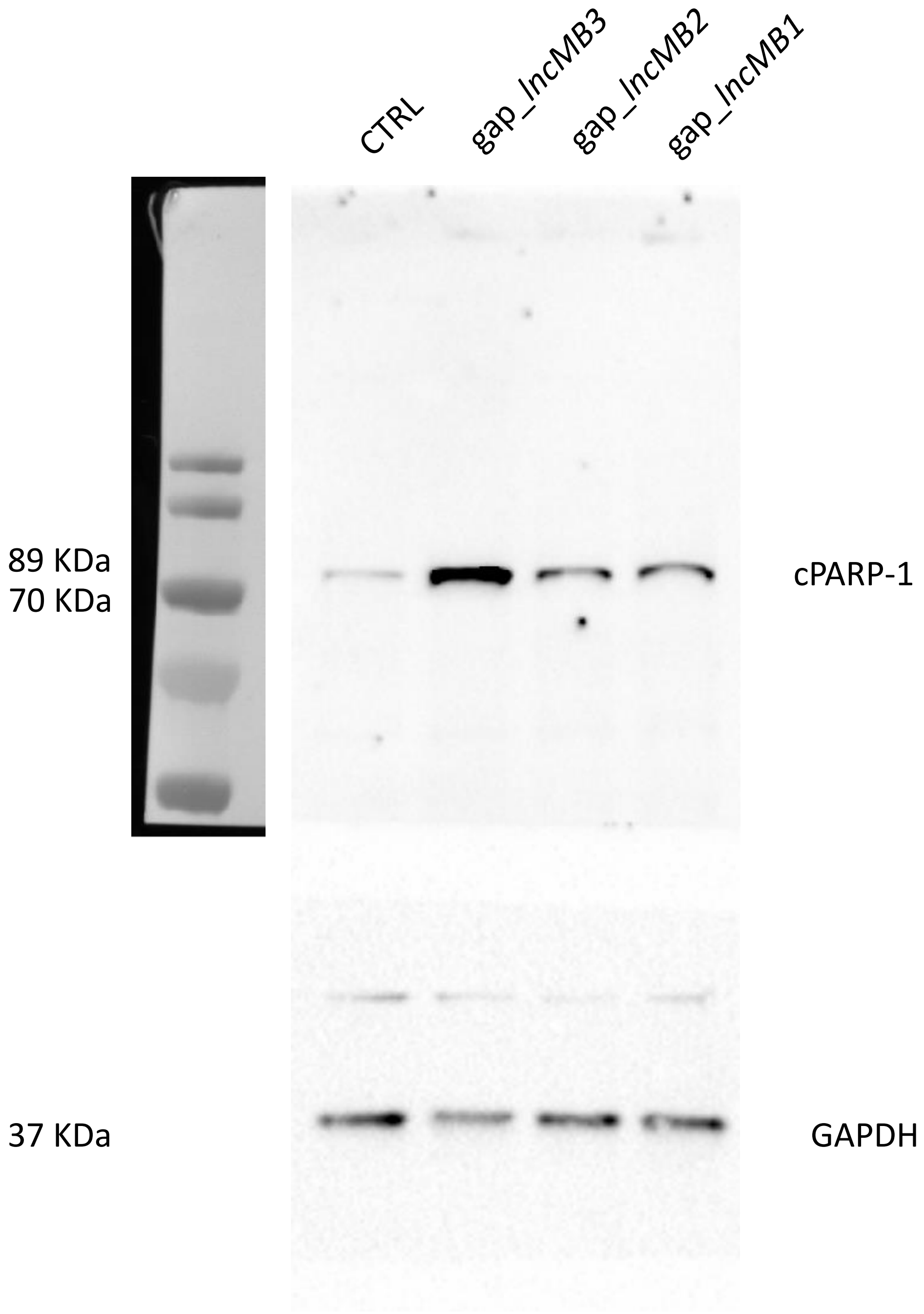


| | GAPDH | cPARP-1 | cPARP/GAPDH | FC cPARP-1 |
|---|---|---|---|---|
| gapCTRL | 2499192 | 551369 | 0.2206189 | 1 |
| gapAC010998.3 | 1360408 | 6993176 | 5.14049903 | 23.30036 |
| gapAC091182.1 | 2030440 | 1981671 | 0.97598107 | 4.423832 |
| gapRHOT1-AS | 2098923 | 2218623 | 1.05702925 | 4.7912 |

**Fig. 10**

**C**

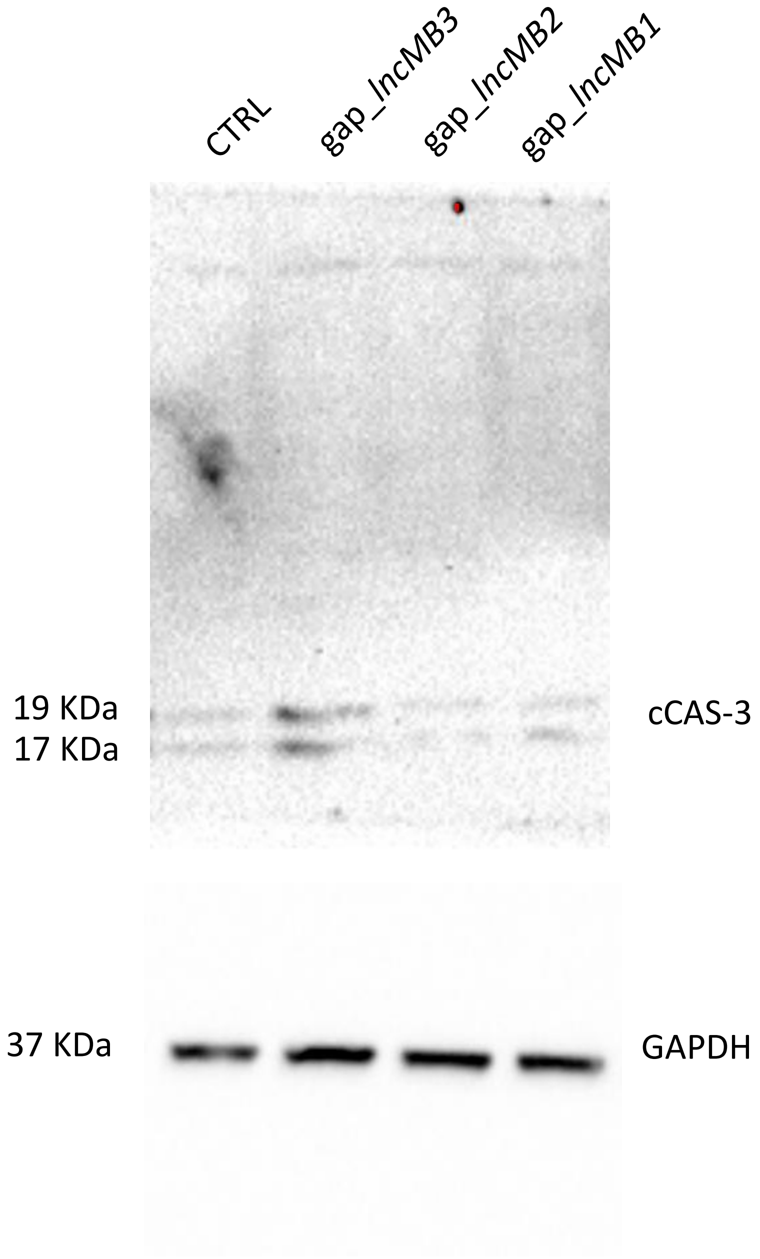


| | GAPDH | c-Cas3 | c-Cas3/GAPDH | FC cCas-3 |
|---|---|---|---|---|
| gapCTRL | 1913.695 | 406.087 | 0.21220048 | 1 |
| gap_lncMB3 | 2787.254 | 1712.026 | 0.61423394 | 2.894593 |
| gap_lncMB2 | 2537.508 | 336.25 | 0.1325119 | 0.624466 |
| gap_lncMB1 | 2279.489 | 458.251 | 0.20103234 | 0.94737 |

# Fig. 12

## B

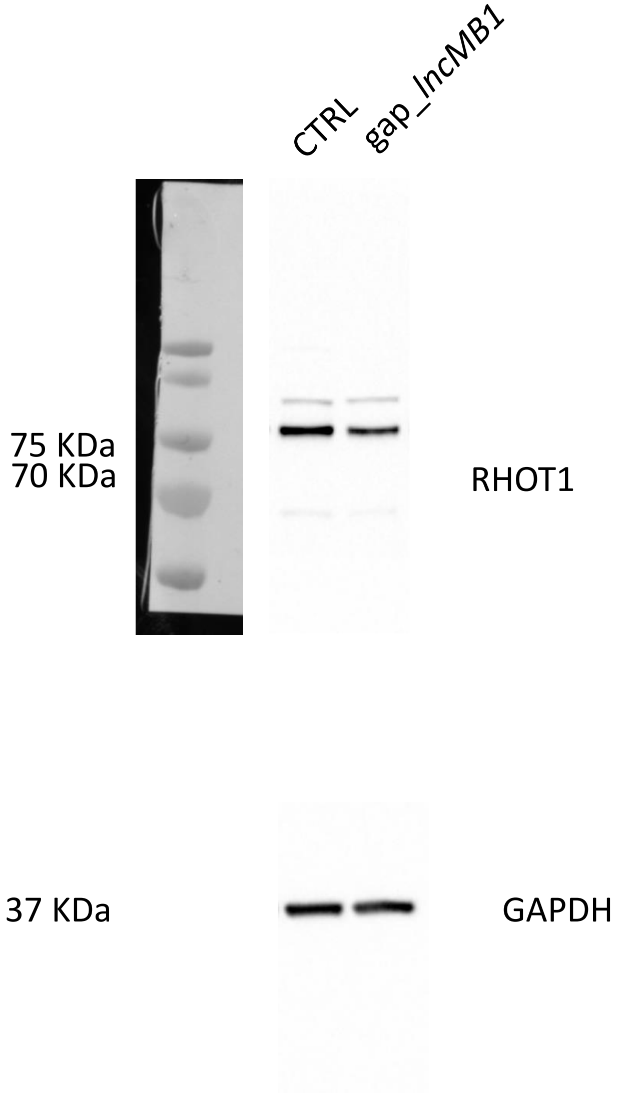


| | GAPDH | RHOT1 | RHOT1/GAPDH | FC RHOT1 |
|---|---|---|---|---|
| CTRL | 2.206154 | 481.787 | 218.383214 | 1 |
| gap_lncMB1 | 2.328482 | 406.322 | 174.500812 | 0.799058 |
