# Supplementary material for: Identification and Functional Characterization of Novel MYC-Regulated Long Noncoding RNAs in Group 3 Medulloblastoma
Source: Cancers (Basel). 2021 Jul 30;13(15):3853. doi: 10.3390/cancers13153853 (PMC8345409; doi:10.3390/cancers13153853)
Supplement: Supplementary file 1 [file cancers-13-03853-s001.zip › Supplementary Materials/Supplementary Figures and Captions.pdf]

cancers

MDPI

Supplementary Material: Identification and Functional Characterization of Novel MYC-Regulated Long Noncoding RNAs in Group 3 Medulloblastoma

Jessica Rea, Annamaria Carissimo, Daniela Trisciuoglio, Barbara Illi, Daniel Picard, Marc Remke, Pietro Laneve and Elisa Caffarelli

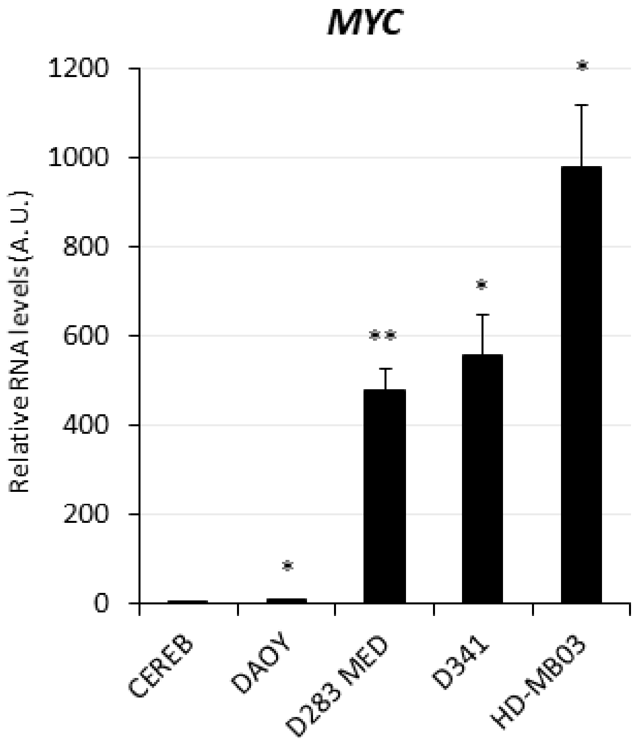

Figure S1. MYC expression in G3- and SHH-derived cell lines compared to cerebellum. qRT-PCR analysis of *MYC* in DAOY, D283 MED, D341, HD-MB03 compared to healthy cerebellum RNA samples (CEREB, set as 1). Data (means ± SEM) are expressed in arbitrary units (A.U.) and are relative to *ATP5O* mRNA levels. $N = 3$, $*p \leq 0.05$, $**p \leq 0.01$ (two-tailed Student's *t*-test).

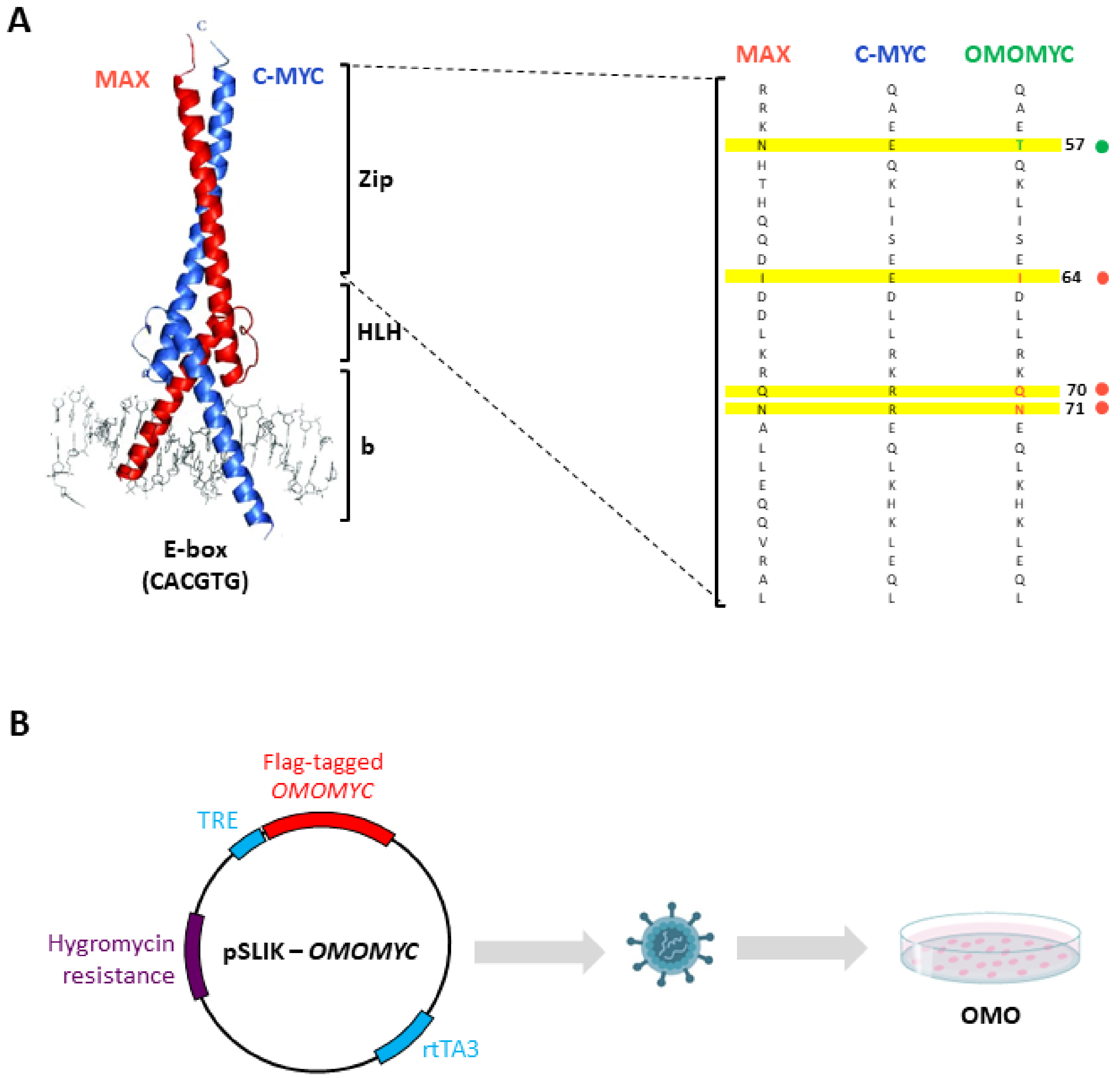

Figure S2. Schematic representation of OMOMYC and pSLIK-OMOMYC construct. (**A**) OMOMYC Structure. (**B**) FLAG tagged OMOMYC cassette is highlighted in red. Hygromycin resistance for transduced cell selection is indicated in blue.

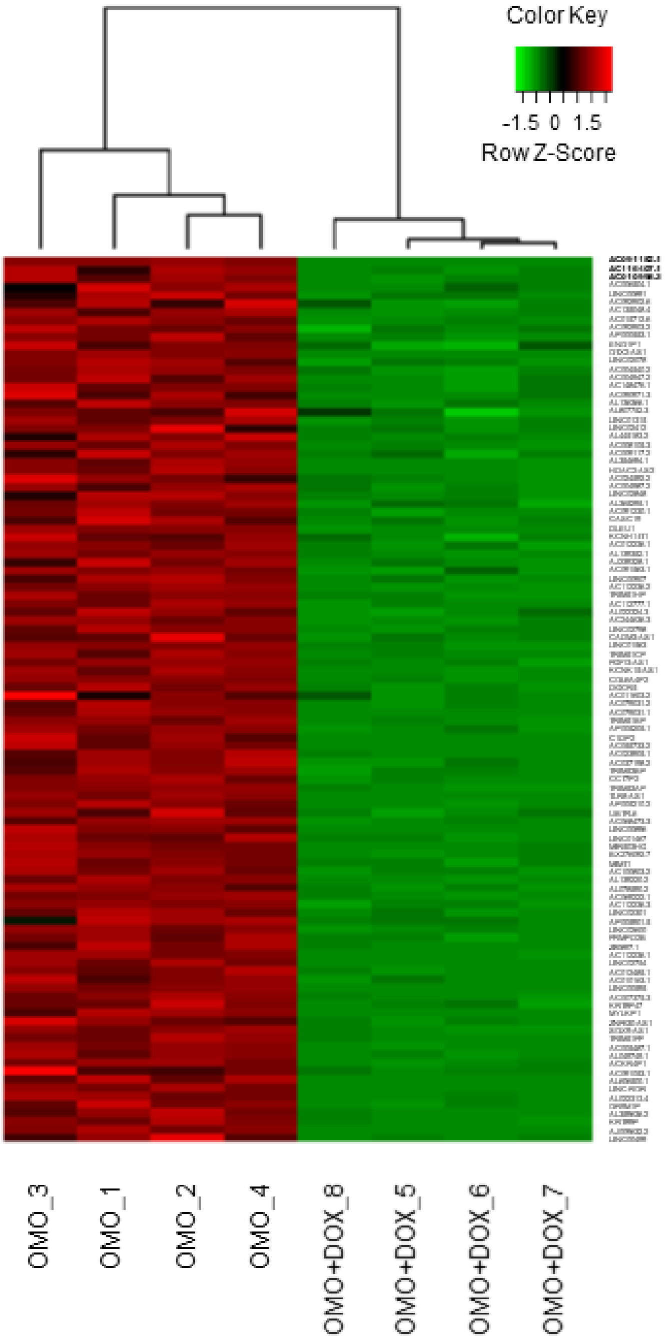

Figure S3. LncRNAs downregulated upon MYC inhibition. Heatmap showing the levels of the 106 lncRNAs downregulated in DOX-treated (72h) D283-OMO cells (OMO + DOX) compared to untreated cells (OMO), along with sample hierarchical clustering. Details as in Figure 2A.

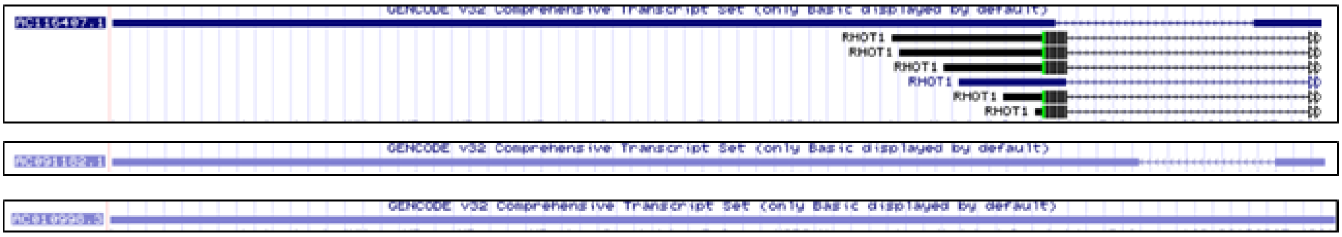

Figure S4. *LncMB1*, *lncMB2* and *lncMB3* genes from UCSC database. UCSC screen shots showing lncMB1, lncMB2 and lncMB3 gene structure.

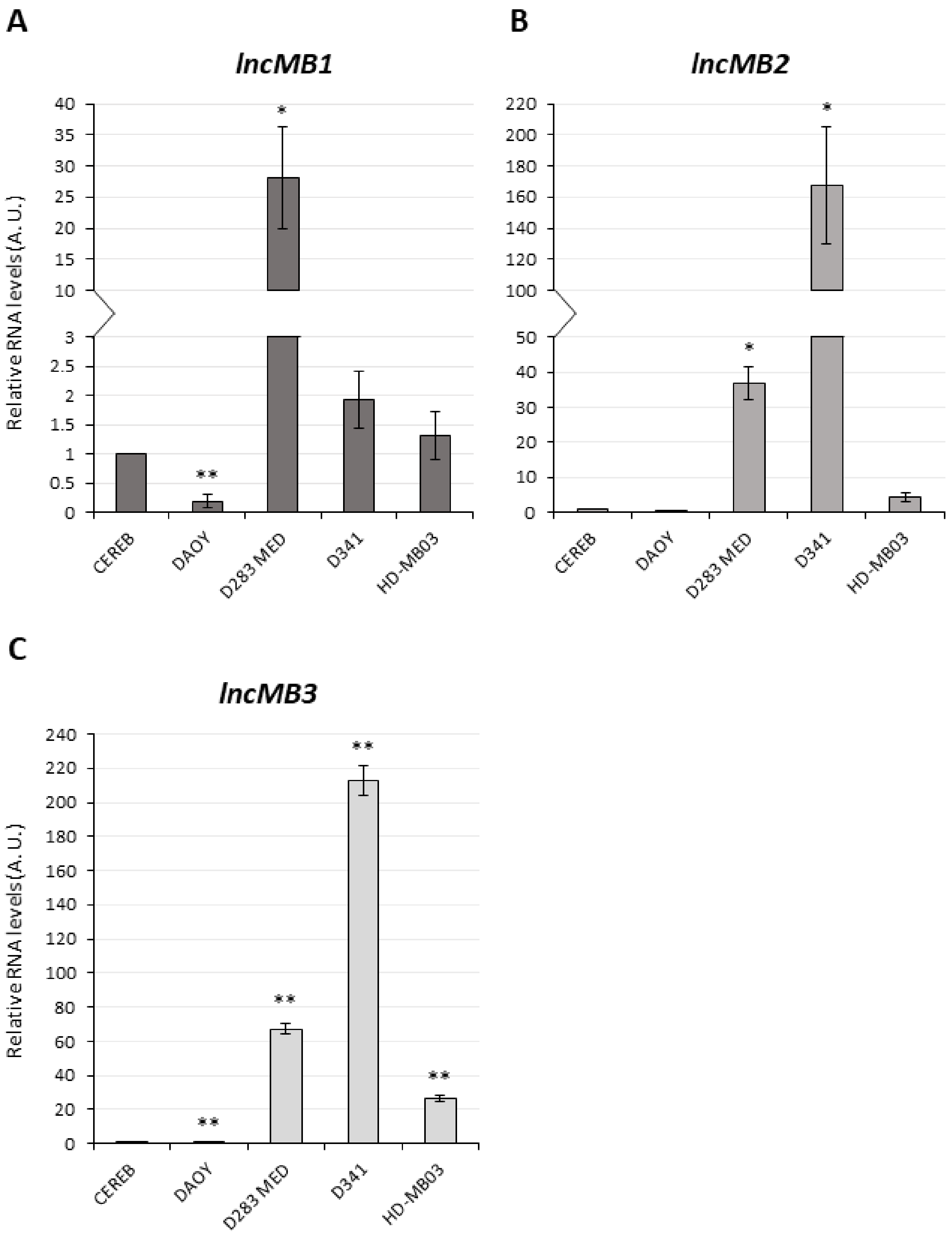

Figure S5. Expression profiles of *lncMB1*, *lncMB2* and *lncMB3* in G3- and SHH-derived cell lines compared to cerebellum. (**A**) qRT-PCR analysis in DAOY, D283 MED, D341, HD-MB03 compared to healthy cerebellum RNA samples (CEREB, set as 1) of *lncMB1*. Data (means ± SEM) are expressed in arbitrary units (A.U.) and are relative to *ATP5O* mRNA levels.N = 3, $*p \leq 0.05$, $**p \leq 0.01$ (two-tailed Student's *t*-test). (**B**) qRT-PCR analysis in DAOY, D283 MED, D341, HD-MB03 compared to healthy cerebellum RNA samples (CEREB, set as 1) of *lncMB2*. $N = 3$, $*p \leq 0.05$ (two-tailed Student's *t*-test). (**C**) qRT-PCR analysis in DAOY, D283 MED, D341, HD-MB03 compared to healthy cerebellum RNA samples (CEREB, set as 1) of *lncMB3*. N= 3, $**p \leq 0.01$.

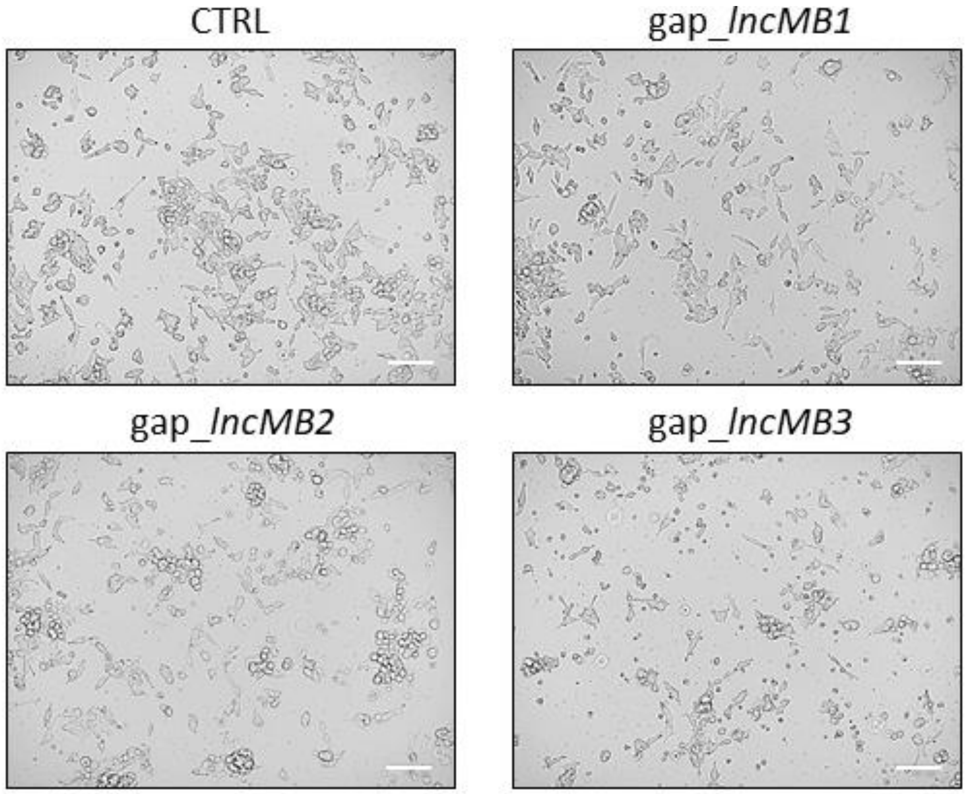

Figure S6. *LncMB1*, *lncMB2* and *lncMB3* knock-down affects DOX-untreated D283-OMO cell morphology. Scale bar: 100 µm. Pictures were taken the day after the last LNA-GapmeR transfection pulse (i.e., day4 after cell seeding).

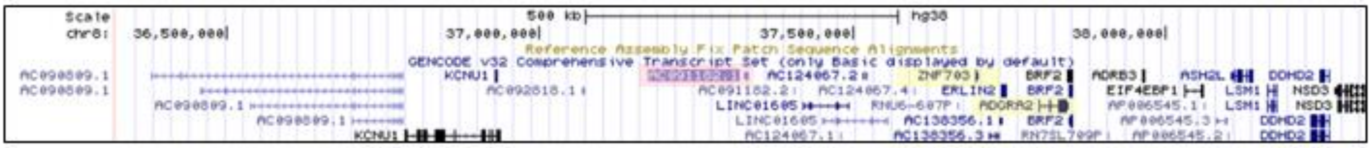

Figure S7. Genomic position of *lncMB2* and its flanking genes from UCSC genome browser. UCSC screenshot showing the chromosomal position and the genomic coordinates of *lncMB2* locus in the human genome. *LncMB2* flanking genes mapping 360 Kb and 460 Kb apart are indicated.

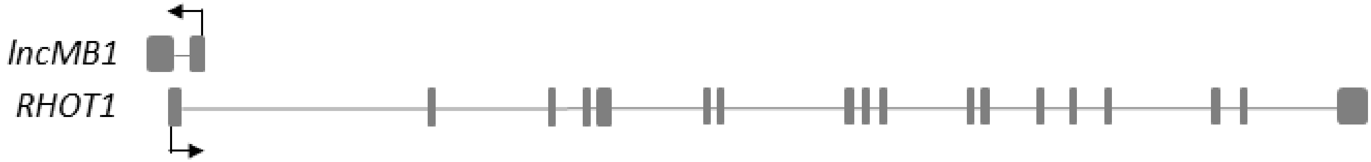

Figure S8. *LncMB1* partially overlaps to *RHOT1* mRNA. Schematic representation of *lncMB1* and *RHOT1* mRNA complementarity. About 36% of *lncMB1* sequence overlaps to *RHOT1* gene.

Table S1 (available in the separate pdf file). List of LNA GapmeRs used in this study.

Table S2(available in the separate pdf file). RNA-Seq read mapping statistics.

Table S3 (available in the separate pdf file). List of oligonucleotides used in this study.

Dataset S1 (available in the separate excel file). Unique gene loci expressed in untreated or DOX-treated D283-OMO cells.

Dataset S2 (available in the separate excel file). Differentially expressed genes FDR < 0.01 $\log_2$FC > |2|.

Dataset S3 (available in the separate excel file). Crossed_CPM + FC_down.

Dataset S4 (available in the separate excel file). Crossed_CPM + FC_up.

Uncropped Western Blot images (available in the separate PDF file). Complete blots from the cropped blots reported throughout the manuscript.

Publisher's Note: MDPI stays neutral with regard to jurisdictional claims in published maps and institutional affiliations.

© 2021 by the authors. Licensee MDPI, Basel, Switzerland. This article is an open access article distributed under the terms and conditions of the Creative Commons Attribution (CC BY) license (http://creativecommons.org/licenses/by/4.0/).
