# Supplementary material for: Identification and Functional Characterization of Novel MYC-Regulated Long Noncoding RNAs in Group 3 Medulloblastoma
Source: Cancers (Basel). 2021 Jul 30;13(15):3853. doi: 10.3390/cancers13153853 (PMC8345409; doi:10.3390/cancers13153853)
Supplement: Supplementary file 1 [file cancers-13-03853-s001.zip › Supplementary Materials/Table S1.pdf]

Table S1

LNA GapmeR name	LNA GapmeR sequence (5'-3')
CTRL	AACACGTGTATACGC
gap_AC116407.1	TAAAAGCACTTAGTCG
gap_AC091182.1	ACGGAGGATTTAATTC
gap_AC010998.3	ACGGTGAGGAGTTAAC
