# Supplementary material for: Identification and Functional Characterization of Novel MYC-Regulated Long Noncoding RNAs in Group 3 Medulloblastoma
Source: Cancers (Basel). 2021 Jul 30;13(15):3853. doi: 10.3390/cancers13153853 (PMC8345409; doi:10.3390/cancers13153853)
Supplement: Supplementary file 1 [file cancers-13-03853-s001.zip › Supplementary Materials/Table S2.pdf]

Table S2

	INPUT READS N°	UNIQUELY MAPPED READS %	MULTI-MAPPING READS %	UNMAPPED READS %
**OMO_1**	60552518	95.12%	4.28%	0.60%
**OMO_2**	64214678	95.06%	4.34%	0.60%
**OMO_3**	60835503	94.81%	4.66%	0.53%
**OMO_4**	55273934	95.24%	4.18%	0.58%
**OMO+Dox_1**	60833164	93.80%	4.03%	2.17%
**OMO+Dox_2**	48271343	93.98%	4.00%	2.02%
**OMO+Dox_3**	61011572	93.62%	4.20%	2.18%
**OMO+Dox_4**	53736082	93.71%	4.17%	2.12%
