# Supplementary material for: Identification and Functional Characterization of Novel MYC-Regulated Long Noncoding RNAs in Group 3 Medulloblastoma
Source: Cancers (Basel). 2021 Jul 30;13(15):3853. doi: 10.3390/cancers13153853 (PMC8345409; doi:10.3390/cancers13153853)
Supplement: Supplementary file 1 [file cancers-13-03853-s001.zip › Supplementary Materials/Table S3.pdf]

Table S3

Gene name	Forward oligo sequence (5'-3')	Reverse oligo sequence (5'-3')
ATP5O	ACTCGGGTTTGACCTACAGC	GGTACTGAAGCATCGCACCT
pre-ATP5O	CTTGGCCTTGACTTAGGAAGC	CAAAGGGAGGAGCTCACAAG
LINC00499	AGCAGTTCATCTCATCTCTCCA	TGTTCTCCGGAAGCTTAGAGTC
AL022313.4	TTTTGGGGTTGGTCCTTACA	GTCTTTGGGGGTCTGTGAAA
AL606500.1	GCCAATTCCAGACACGTTTT	TCATTTGGGGTTCCATTTGT
AC091053.1	AGCGAAACTTCATCCATGCT	GCCGCTTTCTTGAGGTTTTT
AC116407.1	CCGACATGAAGAAAGACGTG	GGGTTCGAAGAGGAAAAGGA
AC091182.1	AGGATTCTGCCGTGAGTGAC	GAGAACACTCCCCTGGTGAA
ACKR4P1	CCCGCTACCTAGGAACATCA	TCTATGGCTCGGCAGAACTT
TRIM51FP	GGACTTCTGGACAGGCTCAG	CAAAAGCCCAATTCCAAGAA
MYLKP1	ATGCAGTTGCCAGATGTCAC	ACCATCAGCACCAACTCCTC
AC007375.3	GAAGCCACCGAGAAATAGGA	GATTCCAAGGTGACCAGTCG
LINC00595	GGTCTCAAACTCCCGTGTTC	CAGTGCTGATGACTCCCTCA
LINC02754	GTCATGATTGAGAGGCAACG	TGACTGATCCCATCACAGGA
Z80897.1	TGGCAGAACAGAATGCTAAACA	TGGGGCTCTTGAAAGTGAAT
LINC02600	TGTCATGTGACCAGGAGGAG	TTCAGCCGCAAGTTACAGAA
AP005901.5	TTTGTAACCTTCGCATTCCA	GATTACAGGCGTGAGCCACT
AC010998.3	GGGCAAAATGTCCAGCTCTA	AGCATGGCTTTGAGAAGAGG
AC112236.3	GGAGAGCTCCAGCAAGGAT	AAATATCAGGCCCTTTGCAG
AC069222.1	CTGGTAGGGGTTCTCCATGA	GAACCTCCACCTGGATTTCA
MIR503HG	TGCTAACTGGAGATGCTGGA	CAGCCTTCCTGAAAGACCAA
AP001626.1	TCACAGACTCCCCCAGAAAC	CAAGGCTGTTCAGGACCAA
FAM238B	GCAACAGAACAAGGGCTCAT	CGAGGGCTGGCTTCTTATC
AC084212.1	GGCACTTGGAGTCCAGACAT	TCTGAGGTCCTTTCCACCAT
AL390961.1	CCCCGTCTGATTCAGGAGT	TTTCTGATCATTGGTTTGATTG
AC108734.4	TTGTGTTAGGCAGCAACAGG	GCAGCCATTCTGTATCGTCA
PAUPAR	GATCGTTTGCAGGGAAGAAA	AGAGGTAAGCCTGGAGCACA
AC103718.1	AAGGACCAAGAGAAAGATGGAG	GCACATGCTGTGAAACTCTCTT
LMCD1-AS1	TCCAGGTGTCCGGTTAAGTC	GCGATCCATTCAGGTCAAAA
AL449106.1	ACAAAGGCGGCTCACTTAAA	ACAGAAATTGGGCGGAAAG
AC245297.1	CCGAGGAGGAAGAAGCAG	ACGGGACCTTTTCACCACTT
CTAGE11P	GGTTGGGGTAAAATCCAGGT	AGGTCCACCACATGTTCCAT
CCDC39	TTGACATTTCAAACCATCCAA	CAACGGCTGGAAAATGAGAT
LINC01151	GCTTCCCACTGGAAAATGGT	GTGGAGAAGAAGGCAACAGC
ANAPC1P1	TGATGCTGTTCAAAATGTGC	GTTCCCCCATGTTCAGAGAA
HSPD1P6	CACCGTAAGCCTTTGGTCAT	AAAACCCTGGAGCCTTGACT
AC015660.1	AAGGTTATGCGTGCACCTCT	GCTGATGTTCTCCTCTGGATG
AL162591.2	GGCAGTAACGCACCATCTCT	TACTTGCTTGCGATGACTGG
AL353768.2	AAATACCAAAGGAAACGAGCA	ACTTGTGCTCTGCAATCCTG
CAD	CCATCCCCAGCATTGATCAC	CATGAGCTGCTATGCCGAC
NCL	GACGAAGATGATGAGGATGACG	CAACTTTTGCAGCTTTCTTTCCT
RHOT	TTTGGCTTCGAGCAAGTTTT	TTGTTTTTGGTAGGGACAGTATTTC
ZNF703	GTCCTCCACTCCCGTCAG	GATCTGCGAGCAGGTCTGAG
ADGRA2	GACTCAGGTTCCCTTCTTGCT	GGACTCCCGGAGAATAGAGG
WDR11	CAGGGAGCAGTTCTCTTTGC	TTCAATGGGTTCTTCCTTGG
BMP7_promo	GGGGACTTTTGGACTAGATGG	CTGGCATTTTTCTGCTCCTT

AC116407.1_promo	CGCCTCCTTTCAGACCTTCT	CGCAATCCCTATTGGTCAGT
AC091182.1_promo	GCCCCAGAAAAGAAGACTCA	CCGTAGCTGCAGATGAAATG
AC010998.3_promo	GCTGGAACAGACTAAGGCACA	TTCAGGACAGGAATGGGTCT
